# Self-reported total sitting time on a non-working day is associated with blunted flow-mediated vasodilation and blunted nitroglycerine-induced vasodilation

**DOI:** 10.1038/s41598-022-10242-8

**Published:** 2022-04-16

**Authors:** Takayuki Yamaji, Takahiro Harada, Yu Hashimoto, Yukiko Nakano, Masato Kajikawa, Kenichi Yoshimura, Kazuaki Chayama, Chikara Goto, Yiming Han, Aya Mizobuchi, Farina Mohamad Yusoff, Shinji Kishimoto, Tatsuya Maruhashi, Ayumu Nakashima, Yukihito Higashi

**Affiliations:** 1 Department of Cardiovascular Medicine, Hiroshima University Graduate School of Biomedical Sciences, Hiroshima, Japan; 2 Division of Regeneration and Medicine, Medical Center for Translational and Clinical Research, Hiroshima University Hospital, Hiroshima, Japan; 3 Department of Biostatistics, Medical Center for Translational and Clinical Research, Hiroshima University Hospital, Hiroshima, Japan; 4 Collaborative Research Laboratory of Medical Innovation, Graduate School of Biomedical and Health Sciences, Hiroshima University, Hiroshima, Japan; 5 Department of Rehabilitation, Faculty of General Rehabilitation, Hiroshima International University, Hiroshima, Japan; 6 Department of Cardiovascular Regeneration and Medicine, Research Institute for Radiation Biology and Medicine, Hiroshima University, 1-2-3 Kasumi, Minami-ku, Hiroshima, 734-8551 Japan; 7 Department of Stem Cell Biology and Medicine, Hiroshima University Graduate School of Biomedical Sciences, Hiroshima, Japan

**Keywords:** Cardiology, Health care

## Abstract

We divided the 466 subjects into two groups based on information on sitting time on a non-working day and evaluated flow-mediated vasodilation (FMD) and nitroglycerine-induced vasodilation (NID). FMD was smaller in subjects with sitting time on a non-working day of ≥6 h/day than in subjects with sitting time on a non-working day of <6 h/day (2.5 ± 2.6% vs. 3.7 ± 2.9%; *p* < 0.001). NID was smaller in subjects with sitting time at non-working day of ≥ 8 h/day than in subjects with sitting time on a non-working day of < 8 h/day (10.1 ± 5.6% vs. 11.5 ± 5.0%; *p* = 0.01). After adjustment for confounding factors for vascular function, the odds of having the lowest tertile of FMD was significantly higher in subjects with sitting time on a non-working day of ≥6 h/day than in subjects with sitting time on a non-working day of <6 h/day. The odds of having the lowest tertile of NID was significant higher in subjects with sitting time on a non-working day of ≥ 8 h/day than in subjects with sitting time on a non-working day of < 8 h/day. These findings suggest that prolonged sitting time on a non-working day is associated with blunted FMD and blunted NID.

## Introduction

Sitting is one of the most popular resting positions for humans. We spend a long time in a sitting position. American adults spend an average time of 6.5 h/day in a sitting position obtained by self-reported questionnaire and Japanese adults spend an average time of 7.0 h/day in a sitting position obtained by self-reported questionnaire^1,2^. One study showed median sitting time was 12 h/day in a sitting position obtained by accelerometers^3^. Previous studies have shown that prolonged total sitting time and leisure sitting time are risk factors for diabetes, cancer, cardiovascular disease (CVD) and all-cause mortality, and prolonged total television viewing time is a risk for depressive symptoms^4–9^. On the other hand, occupational sitting time was more weakly associated with cardiometabolic risk factors than was leisure sitting time^10^. It has been shown that individuals with leisure sitting time of ≥6 h/day have a higher risk for mortality than do individuals with sitting time of < 1 h/day^11^. Furthermore, a decrease in sitting time has beneficial effects on glucose metabolism and lipid profile^12^. Therefore, decrease in sitting time and/or increase in active time may contribute to prevention of several diseases including CVD.

Blunted flow-mediated vasodilation (FMD) occurs in the first step of atherosclerosis, and atherosclerosis finally resulting in cardiovascular events^13,14^. FMD in the brachial artery is one of the most popular markers for assessment of endothelial function since FMD is reduced by traditional cardiovascular risk factors and also before the onset of cardiovascular risks. Not only diabetes mellitus but also impaired fasting glucose, impaired glucose tolerance and fasting blood glucose levels of 95–99 mg/dL are risk for blunted FMD^15–20^. FMD is an independent predictor of cardiovascular events^21^ and FMD is increased by life-style modification or pharmacological treatment^22,23^. Although measurement of nitroglycerine-induced vasodilation (NID) is a well-known control test for FMD measurement, it has recently been shown that NID is a marker of an advanced stage of arteriosclerosis^24^.

Several studies have shown that refraining from sedentary behavior, even for short time, improves endothelial function^25,26^. However, there is no detailed information on the relationship of daily sitting time with vascular function including vascular smooth muscle function. Furthermore, it is also still controversial what kind of sitting time (e.g., total sitting time, sitting time on a non-working day, sitting time on a working day and occupational sitting time) is most correlated with vascular function.

Therefore, in the present study, we assessed the relationship of sitting time with vascular function assessed by FMD as a marker of endothelial function and NID as a marker of vascular smooth muscle function.

## Results

### Baseline characteristics of the subjects

The baseline characteristics of the 446 subjects are summarized in Table 1. The mean age of the subjects was 66 ± 12 years. The 446 subjects included 265 men (59.4%). Among the subjects, 396 (88.8%) had hypertension, 295 (66.1%) had dyslipidemia, 101 (22.6%) had diabetes mellitus, 73 (16.4%) had previous CVD and 50 (11.2%) were current smokers. The median sitting time was 6 h/day. The mean FMD value was 3.1 ± 2.8% and the mean NID value was 11.0 ± 5.2%. The lowest tertile of FMD was 1.6% and the lowest tertile of NID was 8.5%. Therefore, we defined blunted FMD as FMD of < 1.6% and we defined blunted NID of < 8.5%.

**Table 1:** Clinical characteristics of subjects with sitting time on a non-working day of <6 h/day and subjects with sitting time on a non-working day of ≥6 h/day.

Variables	Total (n = 446)	< 6 h/day (n = 215)	≥6 h/day (n = 231)	*P* value
Age, year	66 ± 12	64 ± 13	67 ± 12	0.003
Men, n (%)	265 (59.4)	129 (60.0)	136 (58.9)	0.81
Body mass index, kg/m^2^	24.2 ± 3.7	24.0 ± 3.8	24.4 ± 3.6	0.24
Heart rate, bpm	68 ± 11	66 ± 9	70 ± 11	< 0.001
Systolic blood pressure, mmHg	128 ± 16	130 ± 16	126 ± 15	0.009
Diastolic blood pressure, mmHg	77 ± 11	79 ± 12	75 ± 10	< 0.001
Total cholesterol, mg/dL	191 ± 35	191 ± 32	190 ± 38	0.75
Triglycerides, mg/dL	127 ± 68	127 ± 68	126 ± 67	0.88
HDL-C, mg/dL	61 ± 16	60 ± 15	62 ± 16	0.20
LDL-C, mg/dL	107 ± 29	109 ± 27	106 ± 31	0.35
Creatinine, mg/dL	0.90 ± 0.59	0.83 ± 0.20	0.96 ± 0.79	0.02
Glucose, mg/dL	107 ± 23	107 ± 21	107 ± 25	0.91
Hemoglobin A1c, %	5.8 ± 0.8	5.8 ± 0.7	5.8 ± 1.0	0.68
**Medical history, n (%) **				
Hypertension	396 (88.8)	193 (89.8)	203 (87.9)	0.53
Dyslipidemia	295 (66.1)	144 (67.0)	151 (65.4)	0.72
Diabetes mellitus	101 (22.6)	50 (23.3)	51 (22.1)	0.77
CVD	73 (16.4)	48 (22.3)	25 (10.8)	0.001
Current smoker	50 (11.2)	26 (12.1)	24 (10.4)	0.57
**Medication, n (%)**				
Antihypertensive drugs	378 (95.0)	177 (92.7)	201 (97.1)	0.04
Lipid lowering drugs	197 (66.1)	98 (68.1)	99 (64.3)	0.49
Anti-diabetic drugs	62 (13.9)	32 (14.9)	30 (13.0)	0.56

HDL-C indicates high-density lipoprotein cholesterol; LDL-C, low-density lipoprotein cholesterol; CVD, cardiovascular disease.

### Sitting time and FMD

**Figure 1:**
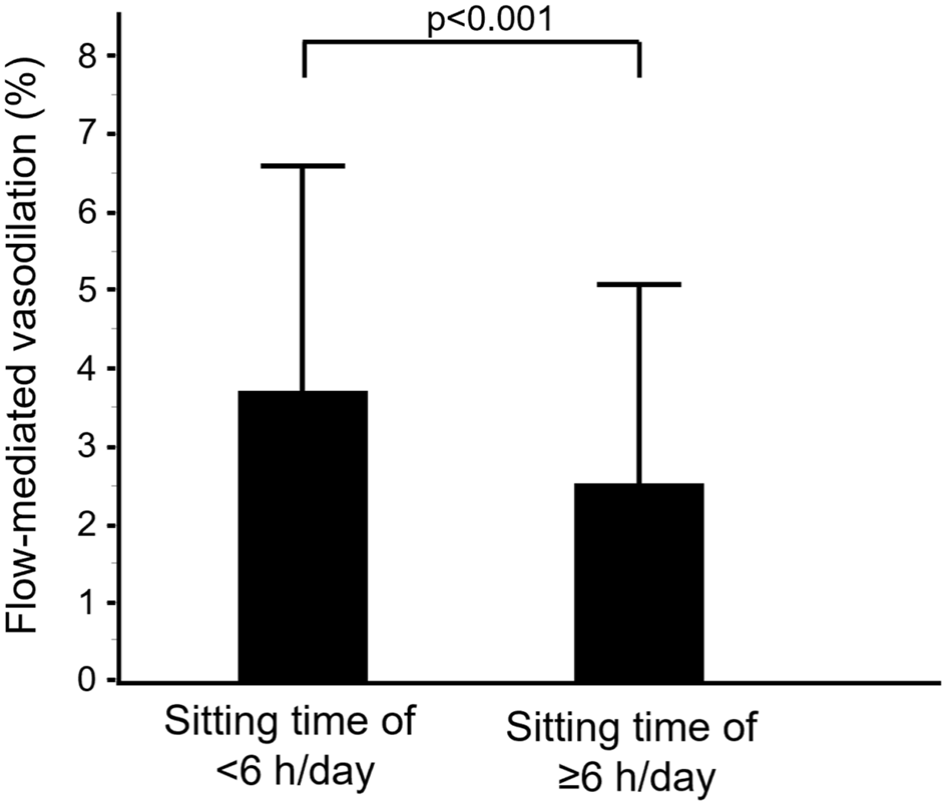
Bar graphs show flow-mediated vasodilation in subjects with sitting time on a non-working day of < 6 h/day and subjects with sitting time on a non-working day of ≥ 6 h/day.

The cut-off value of sitting time on a non-working day from ROC curves for prediction of blunted FMDwas 6 h/day (Figure I in the Data Supplement). Therefore, first, we divided subjects into two groups according to sitting time on a non-working day: subjects with sitting time on a non-working day of < 6 h/day and subjects with sitting time on a non-working day of ≥ 6 h/day. The baseline characteristics of subjects with sitting time on a non-working day of < 6 h/day and subjects with sitting time on a non-working day of ≥ 6 h/day are summarized in Table 1. There were significant differences in age, heart rate, systolic blood pressure, creatinine, diastolic blood pressure, prevalence of CVD, and use of antihypertensive drugs between the two groups (Table 1). FMD values were 3.7 ± 2.9% in the subjects with sitting time on a non-working day of < 6 h/day and 2.5 ± 2.6% in the subjects with sitting time on a non-working day of ≥ 6 h/day (*p* < 0.001) (Fig. 1).

**Figure 2:**
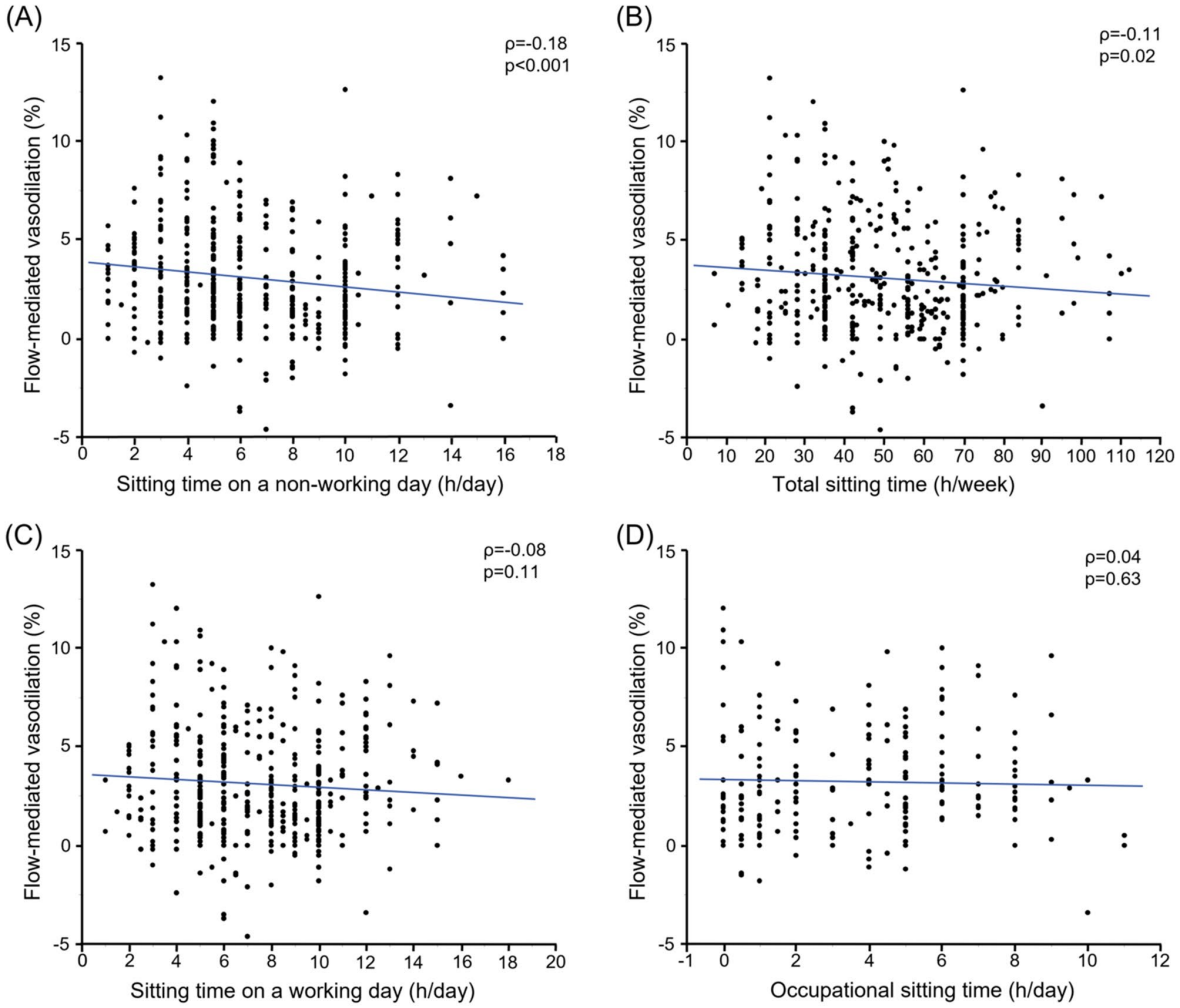
Scatter plots show the relationships of flow-mediated vasodilation with sitting time on a non-working day (**A**), total sitting time (**B**), sitting time on a working day (**C**), and occupational sitting time (**D**).

Univariate analysis revealed that sitting time on a non-working day and total sitting time were correlated with FMD (ρ = − 0.18, *p* < 0.001 and ρ = − 0.11, *p* = 0.02, respectively) (Fig. 2A, B). On the other hand, sitting time on a working day and occupational sitting time were not correlated with FMD (ρ = − 0.08, *p* = 0.11 and ρ = 0.04, *p* = 0.63, respectively) (Fig. 2C, D). We next assessed the ROC curves of sitting time on a non-working day and total sitting time for prediction of blunted FMD. The area under the ROC curve value for sitting time on a non-working day to predict blunted FMD was significantly higher than in that for total sitting time (0.60 vs. 0.55, *p* = 0.006) (Figure II in the Data Supplement). Table I in Data Supplement shows univariate relationships of sitting time with variables. Sitting time on a non-working day was significantly correlated with age (ρ = 0.15, *p* = 0.001), body mass index (ρ = 0.10, *p* = 0.04), heart rate (ρ = 0.15, *p* = 0.002), diastolic blood pressure (ρ = − 0.18, *p* < 0.001), FMD (ρ = − 0.18, *p* < 0.001) and NID (ρ = − 0.13, *p* = 0.009). Total sitting time was significantly correlated with age (ρ = − 0.12, *p* = 0.01), body mass index (ρ = 0.18, *p* < 0.001), heart rate (ρ = 0.16, *p* = 0.001), and FMD (ρ = − 0.11, *p* = 0.02) but was not correlated with NID (ρ = − 0.05, *p* = 0.33). Sitting time on a working day was significantly correlated with age (ρ = − 0.20, *p* < 0.001), body mass index (ρ = 0.21, *p* < 0.001), and heart rate (ρ = 0.15, *p* = 0.001) but was not correlated with FMD (ρ = − 0.08, *p* = 0.11) or NID (ρ = − 0.02, *p* = 0.64). Occupational sitting time was significantly correlated with age (ρ = − 0.36, *p* < 0.001), body mass index (ρ = 0.26, *p* < 0.001), systolic blood pressure (ρ = 0.16, *p* = 0.02), and diastolic blood pressure (ρ = 0.30, *p* < 0.001) but was not correlated with FMD (ρ = 0.03, *p* = 0.63) and NID (ρ = 0.02, *p* = 0.80).

Next, we took the subjects with sitting time on a non-working day of < 6 h/day as a reference and determined whether sitting time on a non-working day was independently associated with blunted FMD by multiple logistic analysis. After adjustments for age, gender, body mass index, presence of hypertension, dyslipidemia, and diabetes mellitus, current smoking, exercise habit, and prevalence of CVD, the odds of blunted FMD were significantly higher in the group with sitting time on a non-working day of ≥ 6 h/day than in the reference group (OR 2.12, 95% CI 1.37–3.29) (Table 2). The adjusted odds ratio (95% CI) for blunted FMD per every one hour/day increase on a non-working day was 1.07 (1.002–1.14) (Table II in the Data Supplement).

**Table 2:** Multivariate analysis of relationships of low tertiles with FMD and sitting time on a non-working day.

Sitting time	Odds ratio (95% Confidence interval); *P* value			
	Unadjusted	Model 1	Model 2	Model 3
<6 h/day	1 (reference)	1 (reference)	1 (reference)	1 (reference)
≥6 h/day	2.37 (1.57–3.58); < 0.001	2.29 (1.49–3.52); < 0.001	2.24 (1.45–3.46); < 0.001	2.12 (1.37–3.29); < 0.001

Model 1; adjusted for age, gender, body mass index, hypertension, dyslipidemia, diabetes mellitus, current smoking.

Model 2; adjusted for age, gender, body mass index, hypertension, dyslipidemia, diabetes mellitus, current smoking, past CVD.

Model 3; adjusted for age, gender, body mass index, hypertension, dyslipidemia, diabetes mellitus, current smoking, past CVD, exercise habit.

FMD indicates flow mediated vasodilation; OR, odds ratio; CI, confidence interval.

Low tertile of FMD indicates less than 1.6%.

### Sitting time and NID

**Figure 3:**
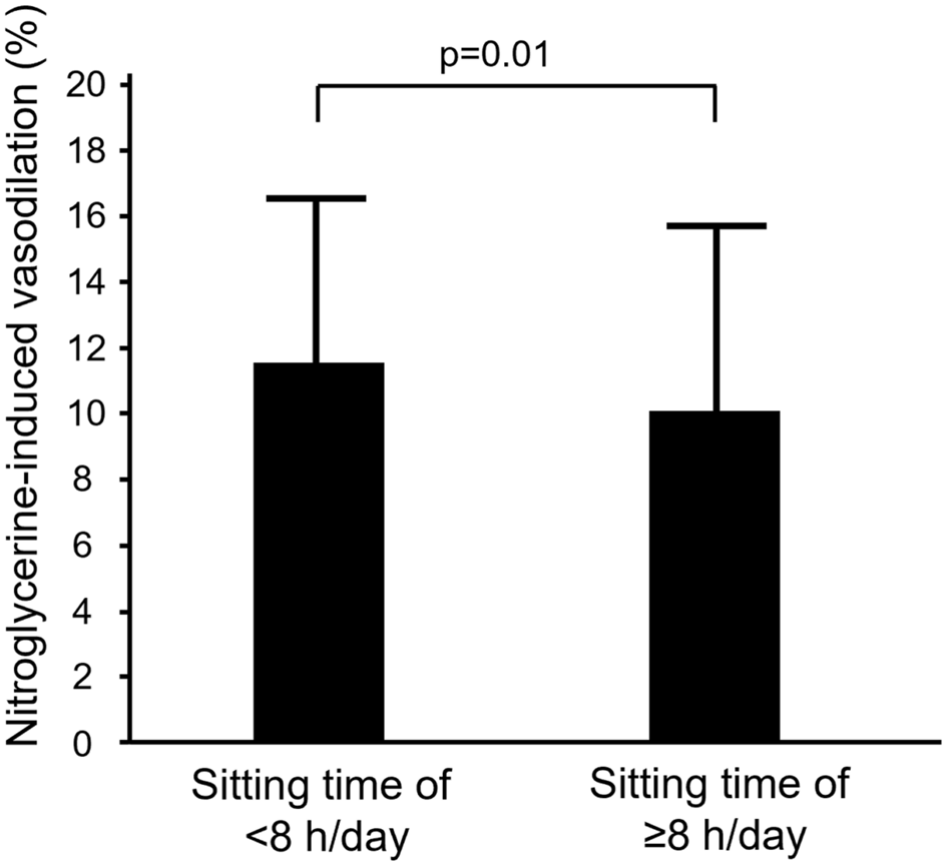
Bar graphs show nitroglycerine-induced vasodilation in subjects with sitting time on a non-working day of < 8 h/day and subjects with sitting time on a non-working day of ≥ 8 h/day.

The baseline characteristics of subjects with sitting time on a non-working day of < 8 h/day and subjects with sitting time on a non-working day of ≥ 8 h/day are summarized in Table 3. There were significant differences in age, gender, body mass index, systolic blood pressure, diastolic blood pressure, and prevalences of hypertension, CVD and current smokers between the two groups (Table 3). NID values were 11.5 ± 5.0% in the subjects with sitting time on a non-working day of < 8 h/day and 10.1 ± 5.6% in the subjects with sitting time on a non-working day of ≥ 8 h/day (*p* = 0.01) (Fig. 3).

**Table 3:** Clinical characteristics of subjects with sitting time on a non-working day of < 8 h/day and subjects with sitting time on a non-working day of ≥ 8 h/day.

Variables	Total (n = 446)	< 8 h/day (n = 286)	≥ 8 h/day (n = 160)	*P* value
Age, year	66 ± 12	64 ± 13	67 ± 11	0.02
Men, n (%)	265 (59.4)	181 (63.3)	84 (52.5)	0.03
Body mass index, kg/m^2^	24.2 ± 3.7	23.9 ± 3.8	24.7 ± 3.4	0.03
Heart rate, bpm	68 ± 11	68 ± 11	69 ± 11	0.21
Systolic blood pressure, mmHg	128 ± 16	129 ± 16	126 ± 15	0.02
Diastolic blood pressure, mmHg	77 ± 11	79 ± 12	74 ± 10	< 0.001
Total cholesterol, mg/dL	191 ± 35	191 ± 34	190 ± 38	0.72
Triglycerides, mg/dL	127 ± 68	124 ± 68	132 ± 68	0.27
HDL-C, mg/dL	61 ± 16	62 ± 15	60 ± 16	0.41
LDL-C, mg/dL	107 ± 29	109 ± 27	105 ± 32	0.25
Creatinine, mg/dL	0.90 ± 0.59	0.86 ± 0.40	0.97 ± 0.83	0.06
Glucose, mg/dL	107 ± 23	107 ± 22	107 ± 25	0.89
Hemoglobin A1c, %	5.8 ± 0.8	5.8 ± 0.8	5.8 ± 0.9	0.63
**Medical history, n (%) **				
Hypertension	396 (88.8)	243 (85.0)	153 (95.6)	0.001
Dyslipidemia	295 (66.1)	188 (65.7)	107 (66.9)	0.81
Diabetes mellitus	101 (22.6)	63 (22.0)	38 (23.8)	0.68
CVD	73 (16.4)	59 (20.6)	14 (8.8)	0.001
Current smoker	50 (11.2)	42 (14.7)	8 (5.0)	0.002
**Medication, n (%)**				
Antihypertensive drugs	378 (95.0)	230 (93.9)	148 (96.7)	0.20
Lipid-lowering drugs	197 (66.1)	128 (68.1)	69 (62.7)	0.35
Antidiabetic drugs	62 (13.9)	39 (13.6)	23 (14.4)	0.83

HDL-C indicates high-density lipoprotein cholesterol; LDL-C, low-density lipoprotein cholesterol; CVD, cardiovascular disease.

**Figure 4:**
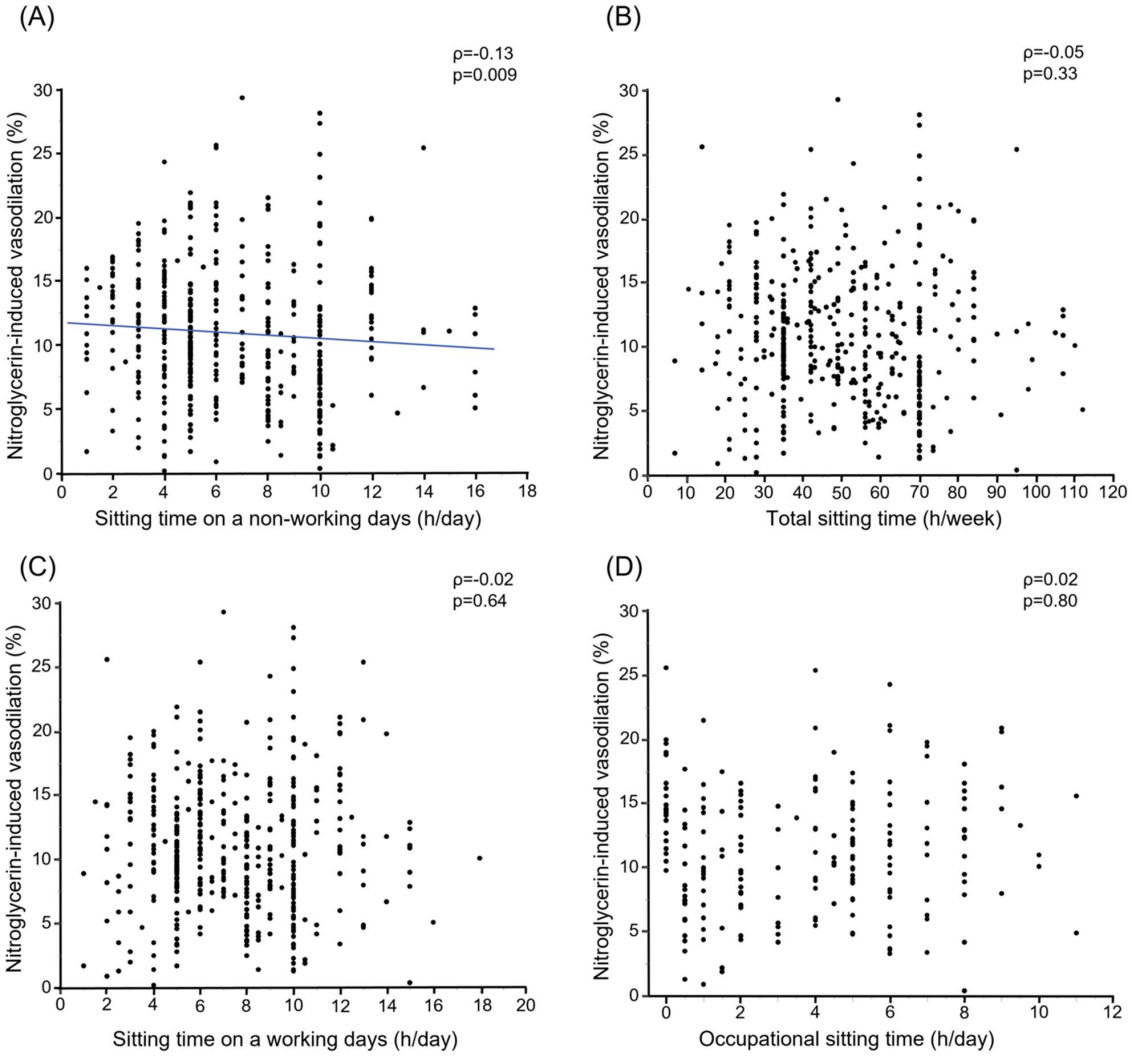
Scatter plots show the relationships of nitroglycerine-induced vasodilation with sitting time on a non-working day (**A**), total sitting time (**B**), sitting time on a working day (**C**), and occupational sitting time (**D**).

Univariate analysis revealed that sitting time on a non-working day was correlated with NID (ρ = − 0.13, *p* = 0.009) (Fig. 4A). On the other hand, total sitting time, sitting time on a working day and occupational sitting time were not correlated with NID (ρ = − 0.05, *p* = 0.33; ρ = − 0.02, *p* = 0.64; and ρ = 0.02, *p* = 0.80, respectively) (Fig. 4B–D). We next assessed the ROC curves of sitting time on a non-working day and total sitting time for prediction of blunted NID. The area under the ROC curve value for sitting time on a non-working day to predict blunted NID was similar between two groups (0.58 vs. 0.55, *p* = 0.08) (Figure IV in the Data Supplement).

Next, we took the subjects with sitting time on a non-working day of < 8 h/day as a reference and determined whether sitting time on a non-working day was independently associated with blunted NID by multiple logistic analysis. After adjustments for confounding factors of cardiovascular risks such as age, gender, body mass index, presence of hypertension, dyslipidemia, diabetes mellitus, current smoking, exercise habit and prevalence of CVD, the odds of blunted NID were significantly higher in the group with sitting time on a non-working day of ≥ 8 h/day than in the reference group (OR 1.76, 95% CI 1.10–2.84) (Table 4). The adjusted odds ratio (95% CI) for blunted NID per every one hour/day increase on a non-working day was 1.07 (0.998–1.15) (Table III in the Data Supplement).

**Table 4:** Multivariate analysis of relationships of low tertiles with NID and sitting time on a non-working day.

Sitting time	Odds ratio (95% Confidence interval); *P* value			
	Unadjusted	Model 1	Model 2	Model 3
< 8 h/day	1 (reference)	1 (reference)	1 (reference)	1 (reference)
≥ 8 h/day	2.08 (1.37–3.17); < 0.001	1.82 (1.16–2.87); 0.01	1.96 (1.23–3.13); 0.005	1.76 (1.10–2.84); 0.02

Model 1; adjusted for age, gender, body mass index, hypertension, dyslipidemia, diabetes mellitus, current smoking.

Model 2; adjusted for age, gender, body mass index, hypertension, dyslipidemia, diabetes mellitus, current smoking, past CVD.

Model 3; adjusted for age, gender, body mass index, hypertension, dyslipidemia, diabetes mellitus, current smoking, past CVD, exercise habit.

NID indicates nitroglycerine-induced vasodilation; OR, odds ratio; CI, confidence interval.

Low tertile of NID indicates less than 8.5%.

### Subgroup analysis related to coronavirus disease 2019 (COVID-19) pandemic

Finally, we evaluated the relationships of sitting time with FMD and NID in subjects who were enrolled before the COVID-19 pandemic and during the COVID-19 pandemic. The baseline characteristics of the 327 subjects enrolled before the COVID-19 pandemic are summarized in Tables IV and V in the Data Supplement. The baseline characteristics of the 119 subjects enrolled during the COVID-19 pandemic are summarized in Tables VI and VII in the Data Supplement. In subjects enrolled before the COVID-19 pandemic, FMD values were 3.2 ± 2.8% in subjects with sitting time on a non-working day of < 6 h/day and 2.0 ± 2.4% in subjects with sitting time on a non-working day of ≥ 6 h/day (*p* < 0.001) (Figure VA in the Data Supplement). NID values were 11.1 ± 4.8% in subjects with sitting time on a non-working day of < 8 h/day and 9.9 ± 5.1% in subjects with sitting time on a non-working day of ≥ 8 h/day (*p* = 0.04) (Figure VB in the Data Supplement). In subjects enrolled during the COVID-19 pandemic, FMD values were 5.2 ± 2.7% in subjects with sitting time on a non-working day of < 6 h/day and 3.6 ± 2.7% in subjects with sitting time on a non-working day of ≥ 6 h/day (*p* = 0.002) (Figure VIA in the Data Supplement). NID values were 12.5 ± 5.2% in subjects with sitting time on a non-working day of < 8 h/day and 10.8 ± 6.7% in subjects with sitting time on a non-working day of ≥ 8 h/day (*p* = 0.14) (Figure VIB in the Data Supplement).

## Discussion

In the present study, we demonstrated for the first time (1) that sitting time on a non-working day was significantly correlated with FMD and NID, while sitting time on a working day and occupational sitting time were not correlated with FMD and NID, (2) that even after adjustments for confounding factors for endothelial function, subjects with sitting time on a non-working day of ≥ 6 h/day had significantly smaller FMD than that in subjects with sitting time on a non-working day of < 6 h/day, and (3) that even after adjustments for confounding factors for vascular smooth muscle function, subjects with sitting time on a non-working day of ≥ 8 h/day had significantly smaller NID than that in subjects with sitting time on a non-working day of < 8 h/day.

First, we defined the sitting time cut-off value for prediction of blunted FMD and blunted NID since the cut-off value of sitting time for vascular function has not yet been established. The subjects with sitting time on a non-working day above the cut-off value of 6 h/day had a significantly higher incidence of blunted FMD than that for subjects with sitting time on a non-working day below the cut-off value after adjustment for confounding factors. In addition, subjects with sitting time on a non-working day above the cut-off value of 8 h/day had a significantly higher incidence of blunted NID than that for subjects with sitting time on a non-working day below the cut-off value after adjustment for confounding factors. Interestingly, the sitting time cut-off value of blunted NID was about two hours longer than that of blunted FMD. It is well known that blunted FMD is the initial step for atherosclerosis and maintains and developments of atherosclerosis and that blunted NID may reflect advanced atherosclerosis. We also previously reported that both FMD and NID were maintained in subjects without cardiovascular risk factors, that FMD was smaller in subjects with cardiovascular risk factors than in subjects without cardiovascular risk factors but that there was no significant difference in NID between subjects with and those without cardiovascular risk factors, and that NID was smaller in patients with CVD than in subjects with cardiovascular risk factors but that FMD was similar in subjects with cardiovascular risk factors and patients with CVD^24^.

Previous studies have shown that not only prolonged sitting activity on a non-working day but also occupational sitting activity and total sitting time per week were associated with all-cause mortality^27,28^. Several investigators showed that leisure-time sedentary behavior (e.g., watching television, using a computer, and sitting in a car) is a predictor of cardiovascular events^4,29,30^. Watching television is the most popular leisure activity and is superior to occupational sitting time for predicting cardiovascular events^29^. It is thought that an increase in sitting activity is sometimes accompanied by unhealthy behavior such as drinking alcohol or eating high-sugar and high-fat snacks, leading to increases in disorders of glucose metabolism and lipid profile that result in cardiovascular events^31^. Frydenlund et al. showed that prolonged sitting time at leisure was associated with cardiovascular risk factors after adjustment for confounding factors^32^. Several studies have shown that refraining from sedentary behavior, even for a short time, improves endothelial function^25,26^. It is expected that augmentation or improvement of endothelial function will prevent future cardiovascular events. Unfortunately, there is no information on the relationship of sitting time with vascular function. In the present study, after adjustment for confounding factors for vascular function, FMD was significantly smaller in subjects with sitting time on a non-working day of ≥ 6 h/day than in subjects with sitting time on a non-working day of < 6 h/day and NID was significantly smaller in subjects with sitting time on a non-working day of ≥ 8 h/day than in subjects with sitting time on a non-working day of < 8 h/day, suggesting that sitting time on a non-working day reflects vascular function.

There are several possible mechanisms by which prolonged sitting time has harmful effects on endothelial function and vascular smooth muscle function. First, shear rates in the leg vasculature are decreased in the supine or seated position compared with those in a standing position^33^. Decreased shear rate (shear stress) leads to an increase in blood viscosity^34^ and activation of inflammatory markers and coagulation markers^34^, resulting in reduction of NO production through a decrease in endothelial NO synthase gene expression^35^. Second, previous studies showed harmful effects of prolonged sitting time on cardiac risk factors including weight, blood pressure, serum blood glucose level, low-density lipoprotein cholesterol levels, waist circumference, and levels of triglycerides and high-density lipoprotein cholesterol^36–40^. Third, it is well known that an increase in muscle sympathetic nerve activity activates vasoconstrictors and leads to blunted FMD and blunted NID^41^. Appropriate physical activity such as aerobic exercise has beneficial effects on sympathetic nerve activity and vascular function. Sverrisdóttir et al. showed that an increase in physical activity is related to a decrease in sympathetic nerve activity and better endothelial function assessed by reactive hyperemia index in healthy subjects^41^. These findings suggest that a decrease in sitting time improves or augments vascular function through a decrease in sympathetic nerve activity.

This study has some limitations. Although this cross-sectional study did not establish the causality between sitting time on a non-working day and endothelial function and vascular smooth muscle function, this study showed the possibility of an association of sitting time on a non-working day with vascular dysfunction including blunted FMD and blunted NID. Second, information on daily behavior including sitting time was obtained from self-administered questionnaires. Measurement of sitting time by an accelerometer would enable more specific conclusions concerning the role of sitting time in endothelial function and vascular smooth muscle function to be drawn. However, IPAQ is one of the most popular tools for assessing daily activity^42,43^. Third, in the present study, we did not have information on inflammatory markers and oxidative stress. Assessment of these markers would enable more specific conclusions concerning the role of sitting time in vascular function to be drawn. Fourth, we did not have information on social and economical factors. Measurement of social or economical factors would enable more specific conclusions concerning the role of sitting time in endothelial function and vascular smooth muscle function to be drawn. Fifth, a total of 119 subjects were enrolled during the COVID-19 pandemic. We cannot deny the possibility that the conditions of the COVID-19 pandemic affected vascular function.

## Conclusions

In conclusions, prolonged sitting time on a non-working day was associated with blunted FMD and blunted NID. A decrease in sitting time may be a target for the prevention of cardiovascular events through improvement or maintenance of FMD and NID.

## Methods

### Study design and participants

This study was a cross-sectional study. The subjects fasted overnight and abstained from drinking alcohol, smoking, and taking caffeine and antioxidant vitamins for at least 12 h before the study. The participants were kept in the supine position in a quiet, dark, air-conditioned room (constant temperature of 22–25 °C) throughout the study. A 23-gauge polyethylene catheter was inserted into the deep antecubital vein to obtain blood samples. After they lay in the supine position for 30 min, FMD and NID were measured. The observers were blinded to the form of examination. A total of 446 subjects who underwent a health checkup at Hiroshima University between January 2016 and October 2020 were recruited in this study. Subjects aged 20–89 years, both sexes, regardless of abnormalities of blood parameters (e.g., lipid profile, glucose metabolism, and renal function), and taking medications such as antihypertensive drugs, lipid-lowering drugs, and anti-diabetic drugs were included. Subjects with severe chronic heart failure (New York Heart Association level of more than III), subjects who were being treated with nitrate, subjects without information on sitting time, and subjects with a gait disorder were excluded^44^. Hypertension was defined as the use of antihypertensive drugs or systolic blood pressure of more than 140 mmHg or diastolic blood pressure of more than 90 mmHg measured in a sitting position on at least 3 occasions. Dyslipidemia was defined according to the third report of the National Cholesterol Education Program^45^. Diabetes mellitus was defined according to the American Diabetes Association recommendation^46^. Smokers were defined as those who were current smokers. CVD was defined as coronary heart disease and cerebrovascular disease. Coronary heart disease included angina pectoris, prior myocardial infarction, and unstable angina^44^. Cerebrovascular disease included ischemic stroke, hemorrhagic stroke, and transient ischemic attack^44^. All methods were performed in accordance with the relevant guidelines and regulations. The Ethics Committee of Hiroshima University approved the study protocol. Written informed consent for participation in this study was obtained from all participants. The protocol was registered in the University Hospital Medical Information Network Clinical Trials Registry (UMIN000003409).

### Evaluation of sitting time and daily activity

All information on physical activity and sitting time was obtained by the self-reported modified International Physical Activity Questionnaire (IPAQ)^47^. This questionnaire consists of questions on exercise habit, exercise for more than 10 min (times/week), duration of exercise time (hours), total exercise time (h/week), number of working days in a week, occupational sitting time (h/day), sitting time on a working day (h/day), and sitting time on a non-working day (h/day)^47^. According to information provided in the questionnaire, we calculated the duration of exercise and total sitting time (h/week).

### Sitting time and vascular function assessed by FMD and NID

First, we assessed the relationship of sitting time with endothelial function assessed by measurement of FMD, an index of endothelium-dependent vasodilation, in 446 subjects. We divided the subjects into two groups based on information on sitting time on a non-working day and assessed vascular function: subjects with sitting time of < 6 h/day and subjects with sitting time of ≥ 6 h/day. Multivariate regression analysis was performed to identify independent variables associated with blunted FMD.

Next, we assessed the relationship of sitting time with vascular smooth muscle function assessed by measurement of NID as an index of endothelium-independent vasodilation. We divided the subjects into two groups based on information on sitting time on a non-working day: subjects with sitting time of < 8 h/day and subjects with sitting time of ≥ 8 h/day. Multivariate regression analysis was performed to identify independent variables associated with vascular smooth muscle function.

### Measurements of FMD and NID

A high-resolution linear artery transducer was coupled to computer-assisted analysis software (UNEXEF18G, UNEX Co., Nagoya, Japan) that used an automated edge detection system for measurement of the brachial artery diameter^24^. A blood pressure cuff was placed around the forearm of each subject. The brachial artery was scanned longitudinally 5–10 cm above the elbow. When the clearest B-mode image of the anterior and posterior intimal interfaces between the lumen and vessel wall was obtained, the transducer was held at the same point throughout the scan by using a special probe holder (UNEX Co.) to ensure consistency of the imaging. Depth and gain setting were set to optimize the images of the arterial lumen wall interface. When the tracking gate was placed on the intima, the artery diameter was automatically tracked, and the waveform of diameter changes over the cardiac cycle was displayed in real time using the FMD mode of the tracking system. This allowed the ultrasound images to be optimized at the start of the scan and the transducer position to be adjusted immediately for optimal tracking performance throughout the scan. Pulsed Doppler flow was assessed at baseline and during peak hyperemic flow, which was confirmed to occur within 15 s after cuff deflation. Blood flow velocity was calculated from the color Doppler data and was displayed as a waveform in real time. Baseline longitudinal images of the artery were acquired for 30 s, and then the blood pressure cuff was inflated to 50 mmHg above systolic pressure for 5 min. The longitudinal image of the artery was recorded continuously until 5 min after cuff deflation. Pulsed Doppler velocity signals were obtained for 20 s at baseline and for 10 s immediately after cuff deflation. Changes in brachial artery diameter were immediately expressed as percentage change relative to the vessel diameter before cuff inflation. FMD was automatically calculated as the percentage change in peak vessel diameter from the baseline value. Percentage of FMD [(peak diameter – baseline diameter)/baseline diameter] was used for analysis. Blood flow volume was calculated by multiplying the Doppler flow velocity (corrected for the angle) by heart rate and vessel cross-sectional area (− r^2^). Reactive hyperemia was calculated as the maximum percentage increase in flow after cuff deflation compared with baseline flow^24^.

The response to nitroglycerine was used for assessment of endothelium-independent vasodilation. After acquiring baseline rest images for 30 s, a sublingual tablet (nitroglycerine, 75 µg) was given and imaging of the artery was done continuously for 5 min. NID was automatically calculated as a percentage change in peak vessel diameter from the baseline. Percentage of NID [(peak diameter − baseline diameter)/baseline diameter] was used for analysis. Inter- and intra-coefficients of variation for the brachial artery diameter were 1.6% and 1.4%, respectively, in our laboratory.

### Statistical analysis

Results are presented as means ± SD. All reported probability values were 2-sided, and a probability value of < 0.05 was considered statistically significant. Categorical values were compared by means of the chi-square test. Continuous variables were compared by using Student’s *t* test. The relationships among FMD, NID, sitting time (sitting time on a non-working day, total sitting time, sitting time on a working day, occupational sitting time) and variables were determined by Spearman’s correlation analysis. Receiver-operator characteristic (ROC) curve analysis was performed to assess the sensitivity and specificity of sitting time for predicting blunted FMD and blunted NID. Cut-off values for sitting time on a non-working day were determined according to the highest Youden index from the receiver-operator characteristic curves for predicting blunted FMD and blunted NID. The area under the ROC curves value of sitting time on a non-working day and total sitting time for prediction of blunted FMD and blunted NID was assessed. The cut-off value of sitting time on a non-working day from ROC curves for prediction of blunted FMD assessed by FMD was 6 h/day (Figure I in the Data Supplement). Therefore, first, we divided subjects into two groups according to sitting time on a non-working day: subjects with sitting time on a non-working day of < 6 h/day and subjects with sitting time on a non-working day of ≥ 6 h/day. The cut-off value of sitting time on a non-working day from ROC curves for prediction of blunted NID assessed by NID was 8 h/day (Figure III in the Data Supplement). Therefore, first, we divided subjects into two groups according to sitting time on a non-working day: subjects with sitting time on a non-working day of < 8 h/day and subjects with sitting time on a non-working day of ≥ 8 h/day. Multivariate logistic regression analysis was performed to identify independent variables associated with lower tertiles of FMD (< 1.6%) and NID (< 8.5%). Age, gender, body mass index, current smoking, presence of hypertension, presence of dyslipidemia, and presence of diabetes mellitus were entered into the multivariate logistic regression analysis. All data were processed using JMP Pro. Ver 14.0 software (SAS Institute, Cary, NC, USA).

## Supplementary Information

Supplementary Information.
