# Supplementary material for: Self-reported total sitting time on a non-working day is associated with blunted flow-mediated vasodilation and blunted nitroglycerine-induced vasodilation
Source: Sci Rep. 2022 Apr 16;12:6366. doi: 10.1038/s41598-022-10242-8 (PMC9012897; doi:10.1038/s41598-022-10242-8)
Supplement: Supplementary file 1 — Supplementary Information. [file 41598_2022_10242_MOESM1_ESM.docx]

**Supplemental Data**

**Self-reported Total Sitting Time on A Non-working Day Is Associated with** **Blunted Flow-mediated Vasodilation and Blunted Nitroglycerine-induced Vasodilation**

Brief title: Sitting time and vascular function

Takayuki Yamaji, MD;^1^ Takahiro Harada, MD;^1^ Yu Hashimoto, MD;^1^ Yukiko Nakano, MD, PhD;^1^ Masato Kajikawa, MD, PhD;^2^ Kenichi Yoshimura, PhD;^2,3^ Kazuaki Chayama, MD, PhD;^4^ Chikara Goto, PhD;^5^ Yiming Han, MS;^6^ Aya Mizobuchi, MS;^6^ Farina Mohamad Yusoff, MD;^6^ Shinji Kishimoto, MD, PhD;^6^ Tatsuya Maruhashi, MD, PhD;^6^ Ayumu Nakashima, MD, PhD;^7^

Yukihito Higashi, MD, PhD, FAHA^2,6^

^1^Department of Cardiovascular Medicine, Hiroshima University Graduate School of Biomedical Sciences, Hiroshima, Japan

^2^Division of Regeneration and Medicine, Medical Center for Translational and Clinical Research, Hiroshima University Hospital, Hiroshima, Japan

^3^Department of Biostatistics, Medical Center for Translational and Clinical Research, Hiroshima University Hospital, Hiroshima, Japan

^4^ Collaborative Research Laboratory of Medical Innovation, Graduate School of Biomedical and Health Sciences, Hiroshima University, Hiroshima, Japan.

^5^Department of Rehabilitation, Faculty of General Rehabilitation, Hiroshima International University, Hiroshima, Japan

^6^Department of Cardiovascular Regeneration and Medicine, Research Institute for Radiation Biology and Medicine, Hiroshima University, Hiroshima, Japan

^7^Department of Stem Cell Biology and Medicine, Hiroshima University Graduate School of Biomedical Sciences, Hiroshima, Japan

Address for correspondence: Yukihito Higashi, MD, PhD, FAHA

Department of Cardiovascular Regeneration and Medicine,

Research Institute for Radiation Biology and Medicine, Hiroshima University

1-2-3 Kasumi, Minami-ku, Hiroshima 734-8551, Japan

Phone: +81-82-257-5831 Fax: +81-82-257-5831

E-mail: [yhigashi@hiroshima-u.ac.jp](mailto:yhigashi@hiroshima-u.ac.jp)

**Supplemental Table**

**Table I.** Univariate Analysis of Relationships of Sitting Time with Variables

| Variables | Sitting time on a non-working day | | Total sitting time | | Sitting time on a working day | | Occupational sitting time | |
| --- | --- | --- | --- | --- | --- | --- | --- | --- |
|  | ρ | P value | ρ | P value | ρ | P value | ρ | P value |
| Age, yr | 0.15 | 0.001 | -0.12 | 0.01 | -0.20 | <0.001 | -0.36 | <0.001 |
| Body mass index, kg/m^2^ | 0.10 | 0.04 | 0.18 | <0.001 | 0.21 | <0.001 | 0.26 | <0.001 |
| Heart rate, bpm | 0.15 | 0.002 | 0.16 | 0.001 | 0.15 | 0.001 | 0.13 | 0.06 |
| Systolic blood pressure, mmHg | -0.09 | 0.05 | -0.02 | 0.73 | <0.001 | 1.00 | 0.16 | 0.02 |
| Diastolic blood pressure, mmHg | -0.18 | <0.001 | 0.02 | 0.63 | 0.08 | 0.11 | 0.30 | <0.001 |
| Total cholesterol, mg/dL | -0.01 | 0.85 | -0.04 | 0.40 | -0.05 | 0.33 | -0.03 | 0.67 |
| Triglycerides, mg/dL | 0.04 | 0.45 | 0.08 | 0.10 | 0.08 | 0.12 | 0.07 | 0.36 |
| HDL-C, mg/dL | 0.01 | 0.83 | -0.02 | 0.65 | -0.03 | 0.52 | -0.04 | 0.54 |
| LDL-C, mg/dL | -0.06 | 0.26 | -0.05 | 0.30 | -0.04 | 0.39 | 0.05 | 0.48 |
| Glucose, mg/dL | -0.06 | 0.19 | -0.04 | 0.44 | -0.03 | 0.60 | 0.11 | 0.13 |
| FMD, % | -0.18 | <0.001 | -0.11 | 0.02 | -0.08 | 0.11 | 0.03 | 0.63 |
| NID, % | -0.13 | 0.009 | -0.05 | 0.33 | -0.02 | 0.64 | 0.02 | 0.80 |

HDL-C indicates high-density lipoprotein cholesterol; LDL-C, low-density lipoprotein cholesterol; FMD, flow-mediated vasodilation; NID, nitroglycerine-induced vasodilation.

Univariate analysis of the relations among sedentary time and variables (Spearman’s correlation analysis).

**Table II.** Multivariate Analysis of Relationships among Blunted FMD and Sitting Time on A Non-working Day

|  | Low tertiles with FMD | |
| --- | --- | --- |
| Variables | OR (95% CI) | P value |
| Unadjusted | 1.10 (1.04-1.17) | 0.002 |
| Model 1 | 1.09 (1.02-1.16) | 0.006 |
| Model 2 | 1.08 (1.01-1.15) | 0.02 |
| Model 3 | 1.07 (1.002-1.14) | 0.04 |

Model 1: adjusted for age, gender and body mass index

Model 2: adjusted for age, gender and body mass index, hypertension, dyslipidemia, diabetes mellitus and current smoking

Model 3: adjusted for age, gender and body mass index, hypertension, dyslipidemia, diabetes mellitus, current smoking, CVD and exercise habit

FMD indicates flow-mediated vasodilation; OR, odds ratio; CI, confidence interval; CVD, cardiovascular disease.

Low tertiles with FMD indicates less than 1.6%.

**Table III.** Multivariate Analysis of Relationships among Low Tertiles with NID and Sitting Time on A Non-working Day

|  | Low tertiles with NID | |
| --- | --- | --- |
| Variables | OR (95% CI) | P value |
| Unadjusted | 1.08 (1.02-1.15) | 0.01 |
| Model 1 | 1.06 (0.99-1.13) | 0.09 |
| Model 2 | 1.08 (0.99-1.14) | 0.08 |
| Model 3 | 1.07 (0.998-1.15) | 0.06 |

Model 1: adjusted for age, gender and body mass index

Model 2: adjusted for age, gender and body mass index, hypertension, dyslipidemia, diabetes mellitus and current smoking

Model 3: adjusted for age, gender and body mass index, hypertension, dyslipidemia, diabetes mellitus, current smoking, CVD and exercise habit

NID indicates nitroglycerine-induced vasodilation; OR, odds ratio; CI, confidence interval; CVD, cardiovascular disease.

Low tertiles with NID indicates less than 8.5%.

**Table IV****.** Clinical Characteristics of Subjects with Sitting Time on A Non-working Day of <6　h/day and Subjects with Sitting Time on A Non-working Day of ≥6 h/day before the COVID-19 Pandemic.

| Variables | Total  (n=327) | <6　h/day  (n=157) | ≥6　h/day  (n=170) | P value |
| --- | --- | --- | --- | --- |
| Age, yr | 66±12 | 64±13 | 67±11 | 0.02 |
| Men, n (%) | 193 (59.0) | 129 (60.5) | 98 (57.7) | 0.60 |
| Body mass index, kg/m^2^ | 24.1±3.6 | 23.9±3.6 | 24.4±3.6 | 0.22 |
| Heart rate, bpm | 68±11 | 65±10 | 70±12 | <0.001 |
| Systolic blood pressure, mmHg | 127±15 | 131±16 | 124±13 | <0.001 |
| Diastolic blood pressure, mmHg | 77±12 | 79±13 | 74±10 | <0.001 |
| Total cholesterol, mg/dL | 189±35 | 191±31 | 188±38 | 0.61 |
| Triglycerides, mg/dL | 124±64 | 122±63 | 126±66 | 0.57 |
| HDL-C, mg/dL | 61±15 | 61±14 | 61±15 | 0.77 |
| LDL-C, mg/dL | 107±28 | 109±27 | 105±30 | 0.24 |
| Creatinine, mg/dL | 0.87±0.57 | 0.81±0.19 | 0.92±0.77 | 0.09 |
| Glucose, mg/dL | 106±22 | 107±20 | 104±23 | 0.31 |
| Hemoglobin A1c, % | 5.8±0.7 | 5.8±0.7 | 5.8±0.8 | 0.97 |
| Medical history, n (%) |  |  |  |  |
| Hypertension | 296 (90.5) | 142 (90.5) | 154 (90.6) | 0.97 |
| Dyslipidemia | 217 (66.4) | 105 (66.9) | 112 (65.9) | 0.85 |
| Diabetes mellitus | 74 (22.6) | 36 (22.9) | 38 (22.4) | 0.90 |
| CVD | 55 (16.8) | 38 (24.2) | 17 (10.0) | <0.001 |
| Current smoker | 35 (10.7) | 16 (10.2) | 19 (11.2) | 0.77 |
| Medication, n (%) |  |  |  |  |
| Antihypertensive drugs | 289 (96.7) | 134 (94.4) | 155 (98.7) | 0.04 |
| Lipid lowering drugs | 150 (68.5) | 75 (71.4) | 75 (65.8) | 0.37 |
| Anti-diabetic drugs | 45 (13.8) | 23 (14.7) | 22 (12.9) | 0.65 |

HDL-C indicates high-density lipoprotein cholesterol; LDL-C, low-density lipoprotein cholesterol; CVD, cardiovascular disease.

**Table V.** Clinical Characteristics of Subjects with Sitting Time on A Non-working Day of <8 h/day and Subjects with Sitting Time on A Non-working Day of ≥8 h/day before the COVID-19 Pandemic.

| Variables | Total  (n=327) | <8 h/day  (n=208) | ≥8 h/day  (n=119) | P value |
| --- | --- | --- | --- | --- |
| Age, yr | 66±12 | 65±13 | 68±10 | 0.04 |
| Men, n (%) | 193 (59.0) | 133 (63.9) | 60 (50.4) | 0.02 |
| Body mass index, kg/m^2^ | 24.1±3.6 | 23.8±3.8 | 24.7±3.3 | 0.03 |
| Heart rate, bpm | 68±11 | 68±11 | 68±11 | 0.63 |
| Systolic blood pressure, mmHg | 127±15 | 129±15 | 124±13 | 0.001 |
| Diastolic blood pressure, mmHg | 77±12 | 79±12 | 73±9 | <0.001 |
| Total cholesterol, mg/dL | 189±35 | 191±34 | 188±36 | 0.47 |
| Triglycerides, mg/dL | 124±64 | 120±64 | 131±64 | 0.13 |
| HDL-C, mg/dL | 61±15 | 62±15 | 59±14 | 0.06 |
| LDL-C, mg/dL | 107±28 | 109±27 | 103±30 | 0.11 |
| Creatinine, mg/dL | 0.87±0.57 | 0.82±0.20 | 0.95±0.91 | 0.045 |
| Glucose, mg/dL | 106±22 | 106±23 | 104±20 | 0.40 |
| Hemoglobin A1c, % | 5.8±0.7 | 5.8±0.8 | 5.7±0.6 | 0.22 |
| Medical history, n (%) |  |  |  |  |
| Hypertension | 296 (90.5) | 179 (86.1) | 117 (98.3) | <0.001 |
| Dyslipidemia | 217 (66.4) | 136 (65.4) | 81 (68.1) | 0.62 |
| Diabetes mellitus | 74 (22.6) | 44 (21.2) | 30 (25.2) | 0.40 |
| CVD | 55 (16.8) | 44 (21.2) | 11 (9.2) | 0.006 |
| Current smoker | 35 (10.7) | 29 (13.9) | 6 (5.0) | 0.01 |
| Medication, n (%) |  |  |  |  |
| Antihypertensive drugs | 289 (96.7) | 174 (95.6) | 115 (98.2) | 0.21 |
| Lipid lowering drugs | 150 (68.5) | 96 (70.6) | 54 (65.1) | 0.39 |
| Anti-diabetic drugs | 45 (13.8) | 27 (13.0) | 18 (15.1) | 0.59 |

HDL-C indicates high-density lipoprotein cholesterol; LDL-C, low-density lipoprotein cholesterol; CVD, cardiovascular disease.

**Table VI.** Clinical Characteristics of Subjects with Sitting Time on A Non-working Day of <6　h /day and Subjects with Sitting Time on A Non-working Day of ≥6　h /day during the COVID-19 Pandemic.

| Variables | Total  (n=119) | <6　h/day  (n=58) | ≥6　h/day    (n=61) | P value |
| --- | --- | --- | --- | --- |
| Age, yr | 65±13 | 62±13 | 67±14 | 0.06 |
| Men, n (%) | 72 (60.5) | 34 (58.6) | 38 (62.3) | 0.68 |
| Body mass index, kg/m^2^ | 24.4±3.9 | 24.3±4.2 | 24.5±3.7 | 0.76 |
| Heart rate, bpm | 68±9 | 66±9 | 70±9 | 0.03 |
| Systolic blood pressure, mmHg | 129±18 | 128±16 | 131±19 | 0.33 |
| Diastolic blood pressure, mmHg | 79±10 | 80±10 | 78±10 | 0.41 |
| Total cholesterol, mg/dL | 194±36 | 193±33 | 195±39 | 0.84 |
| Triglycerides, mg/dL | 135±76 | 142±81 | 127±72 | 0.31 |
| HDL-C, mg/dL | 62±18 | 59±17 | 65±19 | 0.07 |
| LDL-C, mg/dL | 110±31 | 109±27 | 110±35 | 0.93 |
| Creatinine, mg/dL | 0.97±0.63 | 0.87±0.23 | 1.07±0.84 | 0.08 |
| Glucose, mg/dL | 111±27 | 108±22 | 114±30 | 0.24 |
| Hemoglobin A1c, % | 5.9±1.1 | 5.8±0.7 | 5.9±1.4 | 0.49 |
| Medical history, n (%) |  |  |  |  |
| Hypertension | 100 (84.0) | 51 (87.9) | 49 (80.3) | 0.26 |
| Dyslipidemia | 78 (65.6) | 39 (67.2) | 39 (63.9) | 0.70 |
| Diabetes mellitus | 27 (22.7) | 14 (24.1) | 13 (21.3) | 0.71 |
| CVD | 18 (15.1) | 10 (17.2) | 8 (13.1) | 0.53 |
| Current smoker | 15 (12.6) | 10 (17.2) | 5 (8.2) | 0.14 |
| Medication, n (%) |  |  |  |  |
| Antihypertensive drugs | 89 (89.9) | 43 (87.8) | 46 (92.0) | 0.48 |
| Lipid lowering drugs | 47 (59.5) | 23 (59.0) | 24 (60.0) | 0.93 |
| Anti-diabetic drugs | 17 (14.3) | 9 (15.5) | 8 (13.1) | 0.71 |

HDL-C indicates high-density lipoprotein cholesterol; LDL-C, low-density lipoprotein cholesterol; CVD, cardiovascular disease.

**Table VII.** Clinical Characteristics of Subjects with Sitting Time on A Non-working Day of <8 h/day and Subjects with Sitting Time on A Non-working Day of ≥8 h/day during the COVID-19 Pandemic.

| Variables | Total  (n=119) | <8 h/day  (n=78) | ≥8 h/day  (n=41) | P value |
| --- | --- | --- | --- | --- |
| Age, yr | 65±13 | 63±13 | 67±15 | 0.24 |
| Men, n (%) | 72 (60.5) | 48 (61.5) | 24 (58.5) | 0.75 |
| Body mass index, kg/m^2^ | 24.4±3.9 | 24.2±4.0 | 24.8±3.8 | 0.45 |
| Heart rate, bpm | 68±9 | 67±8 | 70±11 | 0.06 |
| Systolic blood pressure, mmHg | 129±18 | 129±17 | 131±20 | 0.50 |
| Diastolic blood pressure, mmHg | 79±10 | 79±10 | 79±11 | 0.75 |
| Total cholesterol, mg/dL | 194±36 | 193±32 | 197±43 | 0.58 |
| Triglycerides, mg/dL | 135±76 | 136±76 | 133±77 | 0.84 |
| HDL-C, mg/dL | 62±18 | 61±16 | 65±21 | 0.21 |
| LDL-C, mg/dL | 110±31 | 109±28 | 111±37 | 0.75 |
| Creatinine, mg/dL | 0.97±0.63 | 0.95±0.68 | 1.01±0.52 | 0.63 |
| Glucose, mg/dL | 111±27 | 109±21 | 116±35 | 0.15 |
| Hemoglobin A1c, % | 5.9±1.1 | 5.8±0.7 | 6.0±1.7 | 0.47 |
| Medical history, n (%) |  |  |  |  |
| Hypertension | 100 (84.0) | 64 (82.1) | 36 (87.8) | 0.42 |
| Dyslipidemia | 78 (65.6) | 52 (66.7) | 269 (63.4) | 0.72 |
| Diabetes mellitus | 27 (22.7) | 19 (24.4) | 8 (19.5) | 0.55 |
| CVD | 18 (15.1) | 15 (19.2) | 3 (7.3) | 0.09 |
| Current smoker | 15 (12.6) | 13 (16.7) | 2 (4.9) | 0.07 |
| Medication, n (%) |  |  |  |  |
| Antihypertensive drugs | 89 (89.9) | 56 (88.9) | 33 (91.7) | 0.66 |
| Lipid lowering drugs | 47 (59.5) | 32 (61.5) | 15 (55.6) | 0.61 |
| Anti-diabetic drugs | 17 (14.3) | 12 (15.4) | 5 (12.2) | 0.64 |

HDL-C indicates high-density lipoprotein cholesterol; LDL-C, low-density lipoprotein cholesterol; CVD, cardiovascular disease.

**Supplemental Figures**

**Figure I**

**
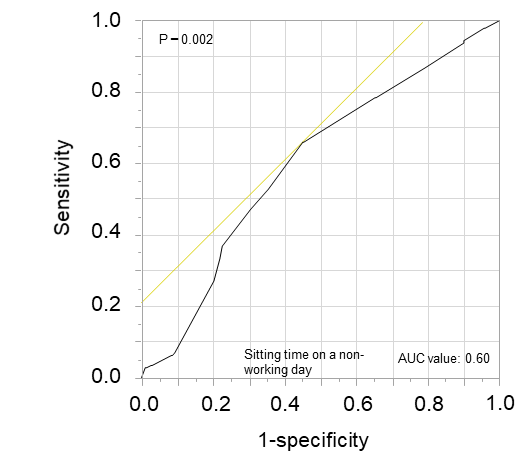
**

**Figure I.** Receiver operating characteristic curves of sitting time on a non-working day for predicting blunted flow-mediated vasodilation.

**Figure II**

**
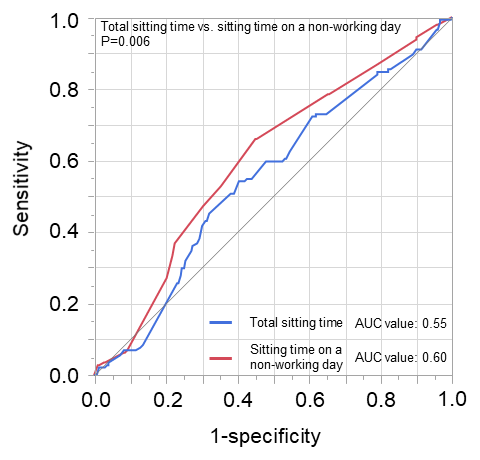
**

**Figure II.** Receiver operating characteristic curves of total sitting time and sitting time on a non-working day for predicting blunted flow-mediated vasodilation.

**Figure III**

**
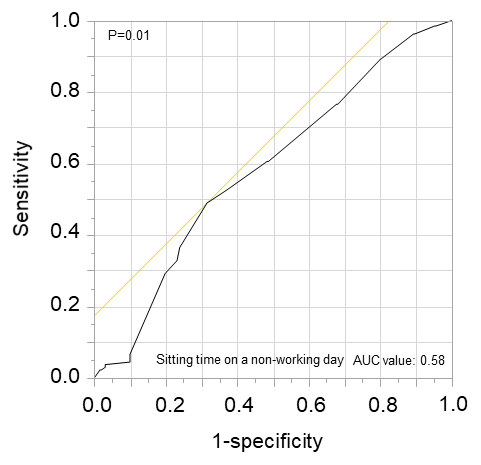
**

**Figure III.** Receiver operating characteristic curves of sitting time on a non-working day for predicting blunted nitroglycerine-induced vasodilation.

**Figure IV**

**
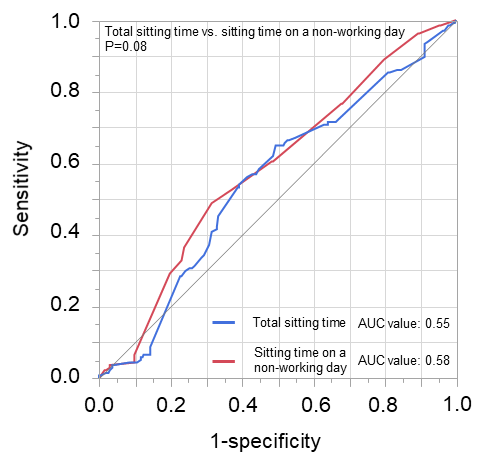
**

**Figure IV** Receiver operating characteristic curves of total sitting time and sitting time on a non-working day for predicting blunted nitroglycerine-induced vasodilation.

**Figure V**

**
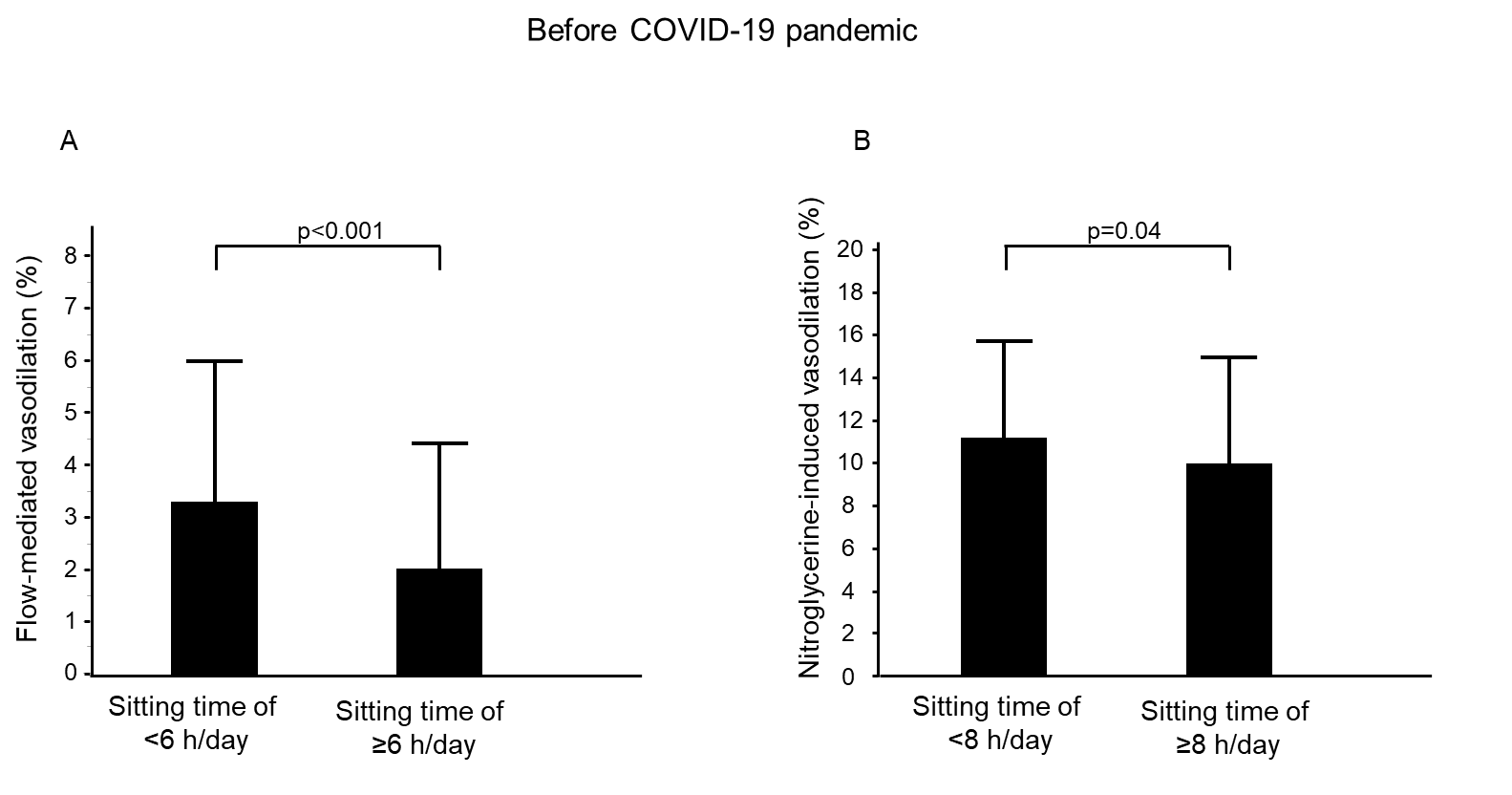
**

**Figure V.** Bar graphs show flow-mediated vasodilation in subjects with sitting time on a non-working day of <6 h/day and subjects with sitting time on a non-working day of ≥6 h/day before the COVID-19 pandemic (A) and nitroglycerine-induced vasodilation in subjects with sitting time on a non-working day of <8 h/day and subjects with sitting time on a non-working day of ≥8 h/day before the COVID-19 pandemic (B).

**Figure VI**

**
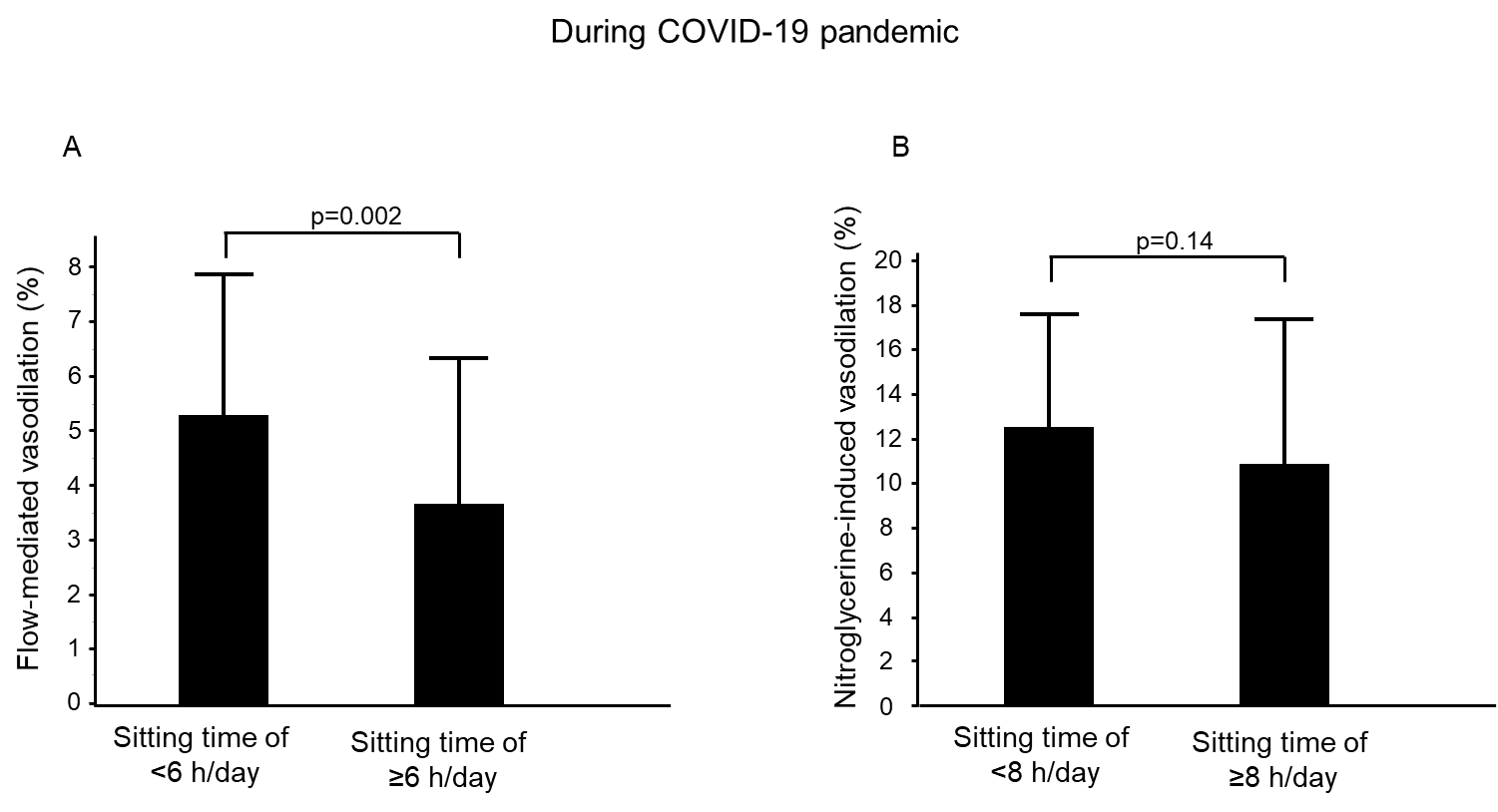
**

**Figure VI.** Bar graphs show flow-mediated vasodilation in subjects with sitting time on a non-working day of <6 h/day and subjects with sitting time on a non-working day of ≥6 h/day during the COVID-19 pandemic (A) and nitroglycerine-induced vasodilation in subjects with sitting time on a non-working day of <8 h/day and subjects with sitting time on a non-working day of ≥8 h/day during the COVID-19 pandemic (B).
